# Local Trends of Antibiotic Prescriptions for Necrotizing Fasciitis Patients in Two Tertiary Care Hospitals in Central Malaysia

**DOI:** 10.3390/antibiotics10091120

**Published:** 2021-09-17

**Authors:** Sanjiv Rampal, Thanusha Ganesan, Narresh Sisubalasingam, Vasantha Kumari Neela, Mehmet Ali Tokgöz, Arun Arunasalam, Mohd Asyraf Hafizuddin Ab Halim, Zulfahrizzat Bin Shamsudin, Suresh Kumar, Ajantha Sinniah

**Affiliations:** 1 Department of Orthopaedic and Traumatology, Faculty of Medicine and Health Sciences, Universiti Putra Malaysia, Seri Kembangan 43400, Malaysia; captdrnash7978@gmail.com (N.S.); vasantha@upm.edu.my (V.K.N.); asyrafhafizuddin05@gmail.com (M.A.H.A.H.); 2 Department of Pharmacology, Faculty of Medicine, University of Malaya, Kuala Lumpur 50603, Malaysia; thanushaganesan@gmail.com; 3 Kecioren Education and Training Hospital, Pınarbaşı Quarter Sanatoryum Street Keçiören, Ankara, Turkey; m.alitokgoz@gmail.com; 4 Urology Department, Hospital Serdang, Ministry of Health of Malaysia, Putrajaya 62590, Malaysia; aa5476@yahoo.com; 5 Department of Orthopaedic Surgery, Hospital Tuanku Ja’afar Seremban, Seremban 70300, Malaysia; zulizzat88@yahoo.com; 6 Centre for Materials Engineering and Regenerative Medicine, Bharath Institute of Higher Education and Research, Chennai 600173, India; sureshkudsc@gmail.com

**Keywords:** necrotizing fasciitis, antibiotics, microorganism, Gram-positive, Gram-negative

## Abstract

Background: Necrotizing fasciitis (NF) is a rapidly progressive inflammatory infection of the soft tissue (also known as the fascia) with a secondary necrosis of the subcutaneous tissues, leading to a systemic inflammatory response syndrome (SIRS), shock and eventually death despite the availability of current medical interventions. The clinical management of this condition is associated with a significant amount of morbidity with a high rate of mortality. The prognosis of the disease is affected by multiple factors, which include the virulence of the causative pathogen, local host immunity, local wound factors and empirical antibiotics used. The local trends in the prescription of empirical antibiotics are often based on clinical practice guidelines (CPG), the distribution of the causative microorganism and the cost-effectiveness of the drug. However, there appears to be a paucity of literature on the empirical antibiotic of choice when dealing with necrotizing fasciitis in the clinical setting. This paper will outline common causative microorganisms and current trends of prescription in two tertiary centres in Central Malaysia. Methods: This was a cross-sectional study using retrospective data of patients treated for NF collected from two tertiary care hospitals (Hospital Seremban and Hospital Ampang) in Central Malaysia. A total of 420 NF patients were identified from the five years of retrospective data obtained from the two hospitals. Results: The top three empirical antibiotics prescribed are ampicillin + sulbactam (*n* = 258; 61.4%), clindamycin (*n* = 55; 13.1%) and ceftazidime (*n* = 41; 9.8%). The selection of the antibiotic significantly impacts the outcome of NF. The top three causative pathogens for NF are *Streptococcus* spp. (*n* = 79; 18.8%), *Pseudomonas aeruginosa* (*n* = 61; 14.5%) and *Staphylococcus* spp. (*n* = 49; 11.7%). The patients who received antibiotics had 0.779 times lower chances of being amputated. Patients with a lower laboratory risk indicator for necrotizing fasciitis (LRINEC) score had 0.934 times lower chances of being amputated. Conclusions: In this study, the most common empirical antibiotic prescribed was ampicillin + sulbactam followed by clindamycin and ceftazidime. The antibiotics prescribed lower the risk of having an amputation and, hence, a better prognosis of the disease. Broad-spectrum empirical antibiotics following surgical debridement reduce the mortality rate of NF.

## 1. Introduction

Necrotizing fasciitis (NF) is a skin and soft tissue infection condition that is rapidly progressive and leads to fulminant tissue necrosis and can be life-threatening if left untreated [1]. Although NF only has an incidence of 0.3 to 15 cases in every 100,000 of the population [2], its mortality rate escalates from 6 to 73% [3,4,5,6,7,8] where the low mortality statistics are attributed to a lower comparative patient age (less than 62 years of age) [9].

The rapid progression of NF is associated with a delayed diagnosis and management due to the initial subtlety of the early signs and symptoms in patients. The risk factors for NF include diabetes mellitus, kidney failure, an advanced age and immunodeficiency [1]. The most common symptoms of NF are oedema (80.1%), pain (79.0%), erythema (73.0%), bullae (25.6%), cutaneous necrosis (24.1%) and subcutaneous emphysema (20.3%) [4,6]. There are four different types of NF with a classification depending on the microbiology, depth of the tissue involvement and location [10].

Type I NF, which is the most common, involves a polymicrobial infection that involves anaerobes more frequently than not. A type II infection is monomicrobial and largely caused by β-haemolytic *streptococcus* (GAS) and occasionally by *S. aureus*. The causative agent for type III is often Gram-negative bacteria and is marine-related and type IV is due to fungal infections such as *Candida.* spp. This paper outlines the most common empirical antibiotics prescribed for the treatment of NF in two tertiary hospitals in Malaysia.

The diagnosis of this disease is predominantly clinical and always requires a high degree of suspicion and therefore initiates a prompt surgical intervention [11]. Laboratory and radiographic tests can be performed upon confirmation of the diagnosis [11]. NF usually begins with a slight inflammation of the soft tissue area that abruptly progresses with fasciitis accompanied by systemic toxicity [11]. An LRINEC is one of the clinical tools used to diagnose NF and was first described by Wong et al. [12]. The tool consists of six different parameters at the time of presentation, which include C-reactive protein, the total white cell count, haemoglobin, serum sodium, creatinine and glucose [13]. Utilising an LRINEC has a potential in dampening the morbidity and mortality rate of NF patients [13].

NF is often mistaken for cellulitis; hence, a high index of suspicion is critical to abstain from morbidity and mortality as NF requires surgical debridement. The distinct signs that differentiate cellulitis from NF include swelling, erythema and severe pain that is not proportionate to the observed lesion. The exacerbation of erythema and skin induration regardless of intravenous (IV) antibiotic use signifies the early stage of NF.

As the disease progresses, blisters, bullae draining haemorrhagic fluid, a violaceous discolouration of the skin and the presence of crepitus are a few of the common signs. Patients will also start manifesting systemic symptoms such as a fever, chills, hypotension, tachycardia and an altered consciousness at the later stage of the disease. However, it is possible for several patients to not demonstrate all of the above symptoms and the possibility of NF should not be excluded due to their absence [11].

Due to its severity, patients should be immediately prescribed broad-spectrum antibiotics as soon as NF is suspected [14]. The choice of antibiotics should be based on a microbiological classification of the disease and the mean duration for an antibiotic treatment for NF is 4–6 weeks [14]. The gold standard of NF management across the globe as per the existing guidelines is an antibiotic treatment following surgical debridement; however, the type of empirical antibiotics prescribed differ from one region to another [8]. Such differences stem from variations in the distribution of pathogens in different parts of the world, which can be attributed to different lifestyles. For instance, most Asian households have wet bathrooms whereas Western cultures prefer a drier environment. Such differences in humidity and damp conditions contribute to the variability in the pathogen type and the recovery period, hence affecting the choice of antibiotics as well [15]. Thus, this study aimed to highlight the practice of antibiotic choice in NF and how it affects the outcome of the condition in two different hospitals in Asia.

## 2. Methodology

### 2.1. Collection of Data

We conducted a retrospective study involving 420 patients at two public tertiary hospitals in Central Malaysia (Hospital Seremban and Hospital Ampang) using electronic dispensing records to review patients diagnosed with NF from January 2014 to December 2018. A review of the clinical records of each patient diagnosed with NF was performed to collect data on the clinical presentation, aetiological agents, type of NF, site of infection and clinical management (antibiotic regime). The sample was collected by two medical doctors and was rechecked by another researcher to identify missing information and outliers. Prior to starting the study, the quality of the research and data were checked through validity and reliability tests.

### 2.2. Inclusion and Exclusion Criteria

Only patients who were clinically and microbiologically diagnosed with NF were included in the study. The other inclusion criteria included an age above 18 and Malaysian. Any patients who were cognitively impaired and referred to the centres were excluded from this study.

### 2.3. Statistical Analysis

The results are presented as mean (standard deviation, SD) or frequencies and percentages. Continuous variables were compared between patients who underwent an amputation and those who did not require an amputation using an unpaired *t*-test and categorical variables with a chi-squared test, except where 25% of the cells had expected counts of 5, in which case the Fisher exact test was used.

*p*-values of 0.05 were regarded as being statistically significant. Significant factors in the univariate analysis were entered into a multiple linear regression model with an amputation as an outcome. A stepwise selection based on the model fit was performed to create the final model. No interaction terms were identified. A population incidence analysis was modelled using a quasi-Poisson regression. The normality of the data was neither assumed nor needed for the inferential analyses. Missing covariate data entailed a case-wise deletion from the analysis set. All tests were carried out at a 5% significance level against two-sided alternatives. Data were analysed with a multivariate statistical analysis using SAS version 9.3 (SAS Institute Inc., Cary, NC, USA).

## 3. Results

The study, which was conducted in two different tertiary hospitals in peninsular Malaysia from 2014–2018, investigated different parameters including gender, race, age group, LRINEC scoring and the type of antibiotics used in NF and their association with the disease outcome. More than half of the patients were male (58.9%) and 66% of patients were of Malay ethnicity followed by Indian and Chinese at 22.9% and 10.5%, respectively (Table 1). The highest cases were recorded in patients between the age group of 50–69, which accounted for 56.7% of the total number of cases.

Ampicillin + sulbactam is the most common empirical treatment to target a wider range of microorganisms. In this retrospective study of NF, ampicillin + sulbactam was prescribed to 258 out of 420 (61.4%) patients (Table 2). Amongst the 124 patients who went through an amputation, 82 (66.1%) were prescribed ampicillin + sulbactam and 176 out of 296 patients with no amputation (59.4%) were being treated with ampicillin + sulbactam, which reiterates that this drug was the first choice of treatment for NF (Table 3). In patients treated with ampicillin + sulbactam, the possibility of superinfections with mycotic or bacterial pathogens should be taken into consideration during therapy [16]. Superinfections include *Pseudomonas* or *Candida* species and given that *Pseudomonas* is one of the main organisms in amputation scenarios, ampicillin + sulbactam’s ineffectiveness could be explained in terms of the resistance of causative organisms to the treatment in cases where patients needed an amputation [16].

Clindamycin has activity against Gram-positive aerobes and anaerobes and selected Gram-negative anaerobes [17]. In this study, clindamycin was the second most used antibiotic with 55 patients being prescribed it (13.1%). Among the 124 patients who went through an amputation, 17 were prescribed clindamycin (13.7%) and 38 out of 296 patients who did not have an amputation were prescribed clindamycin, which accounted for 12.8% (Table 3). Following ampicillin + sulbactam, clindamycin was the second most common empirical treatment for NF given in the two hospitals involved in this study.

Ceftazidime was the third most prescribed antibiotic with about 9.8% of NF patients being prescribed this drug in this study. Ceftazidime belongs to the cephalosporin class of drugs and its mechanism of action involves blocking the bacterial cell wall synthesis [18]. In the cases with no amputation, 10.1% were prescribed ceftazidime whereas among the patients who underwent an amputation, 3.7% (or 11 patients) (Table 3) were given this antibiotic.

In this study, the most common causative Gram-positive and Gram-negative bacteria were *Streptococcus* sp. (*n* = 79) and P. *aeruginosa* (*n* = 61), respectively (Table 2). Given that ampicillin + sulbactam, clindamycin and ceftazidime are not as effective against P. *aeruginosa*, the treatment of patients infected by these organisms might have been rendered ineffective and led to no recovery or amputation in NF involving these organisms [16,17,18].

### 3.1. Study Population and Clinical Characteristics

A total of 420 patients with NF were identified from the five years of retrospective data obtained from the two hospitals. More than half of the NF patients were male (*n* = 246; 58.9%) (Table 1). The number of cases was found to be high in 2017. With a median age of 56.25, the age groups between 50–59 showed the highest number of cases. Among the three major races in Malaysia, Malays followed by Indians accounted for the highest number of NF cases.

Amputation was required in 124/420 (29.5%) NF cases at different levels (Table 4). No significant association was found for gender, race or age with regard to amputation. Patients with an amputation had a significantly higher LRINEC score compared with those patients without an amputation (*p* = 0.016). As expected, patients with an amputation had a significantly longer time of stay in hospital compared with those patients without an amputation (*p* = 0.012).

Among the 420 NF patients, 366 (87.1%) were infected by a monomicrobial organism, 5 (1.2%) had a polymicrobial infection and 49 (11.7%) patients were without any presence of a microorganism. As listed in Table 5, Gram-positive pathogens were detected in 180 (38.3%) NF patients and 149 (31.7%) showed an infection by Gram-negative organisms. The *Streptococcus* species was found to be the most common Gram-positive organism (*n* = 79) and *Pseudomonas aeruginosa* (*n* = 61) was the most frequently encountered Gram-negative organism. Patients who were infected by *Streptococcus* had fewer chances of being amputated and a better recovery (*n* = 65; 82.3%) (Table 5).

For the treatment of NF, ampicillin + sulbactam (61.4%) was commonly prescribed followed by other types of antibiotics (16.4%) (Table 5). A tripartite relation between the causative agent, antibiotic regimen and outcome was investigated to determine the suitable antibiotic for recovery without an amputation. In total, 124 (29.5%) patients had an amputation whereas 296 (70.5%) patients recovered. As shown in Table 2, it was found that both the types of microorganisms (*p* < 0.001) and the types of antibiotics (*p* = 0.231) were statistically significant with amputations. *Streptococcus* had the highest growth (*n* = 79). Among the 420 NF patients, 258 (61.4%) were treated with ampicillin + sulbactam followed by clindamycin (*n* = 55, 13.1%). However, using other antibiotics such as cefuroxime, erythromycin, penicillin, gentamycin, cloxacillin, ceftazidime, imipenem, piperacillin/tazobactam and amoxicillin/clavulanic acid showed a better recovery with a zero amputation rate. Among these antibiotics, cloxacillin was used the most (*n* = 11).

### 3.2. Predictors of the Amputation Rate

A multiple linear regression was used to determine the predictors of the amputation rate. Five variables that should have had a significant association with the rate of amputation were included in the preliminary model. All the variables were analysed using the ‘ENTER’ method and ‘STEPWISE’ method. The final model was obtained using the “ENTER “method because it had the highest *R*^2^ value. The interpretation was made based on the reference groups. From the variables analysed, only the laboratory risk indicator for NF (the LRINEC score) (*p* = 0.009) and the types of antibiotics (*p* = 0.045) were significant. The patients who received antibiotics had 0.779 times lower chances of being amputated. Table 6 shows that patients with a lower LRINEC score had 0.934 times lower chances of being amputated.

## 4. Discussion

NF is a relatively uncommon disease but is often associated with a high mortality and morbidity rate. Immediate surgical debridement and broad-spectrum empiric antibiotics are first-line empirical treatments; however, the paucity of practice guidelines for the best management of NF via antibiotics is one of the gaps aimed to be filled by this study [10].

Most established international guidelines recommend an immediate empirical antibiotic treatment (broad-spectrum antibiotics) to reduce the mortality rate of NF (Table 2). It is suggested by The Infectious Disease Society of America that a bacterial cultural assessment is obtained to aid the selection of antibiotics against the causative pathogens initiated at a targeted therapy for a better outcome of the disease [19]. Similar suggestions to monitor progress and prescribe antibiotics based on cultural findings are also given by the UK’s National Institute for Clinical Excellence (NICE) [20].

The cases showed an upward trend from the 2014 to 2018 with the disease affecting more males than females overall. People aged 50–79 recorded the greatest number of cases with the patients mostly being Malays followed by Indians and Chinese. Gender, race and age, however, did not have a significant correlation with an amputation. The LRINEC scoring and hospital stay were reported to have a significant association with an amputation in NF patients and patients who were prescribed antibiotics had 0.779 times lower risks of going through an amputation, reiterating the importance of an effective antibiotic prescribing practice in the disease. Similar studies in Spain and Kenya have shown a significant correlation between a higher LRINEC score and a poorer outcome of the disease in patients with necrotizing fasciitis [21,22]. Hence, an LRINEC can also be an assisting tool in the antibiotic prescribing practice in NF.

In this study, the most commonly prescribed antibiotic was ampicillin + sulbactam, an injectable combination (IV/IM) of ampicillin sodium and the beta-lactamase inhibitor sulbactam sodium. Ampicillin acts via inhibiting the synthesis of the cell wall mucopeptide and its inhibitory activity is effective against a wide range of bacteria including Gram-positive, Gram-negative, aerobic and anaerobic bacteria. Ampicillin + sulbactam was prescribed for 61.4% of patients in this study as the addition of sulbactam prevents the degradation of ampicillin by beta-lactamase-producing organisms making ampicillin + sulbactam an antibacterial of choice for a broad-spectrum therapy [16]. Its broad-spectrum activity and low cost make it a favourable option of antibiotic in the treatment of NF.

The second most common antibiotic used in this retrospective study was clindamycin with 13.1% of patients receiving the treatment. Clindamycin is also suggested by the Infectious Disease Society of America [23]. Clindamycin belongs to the group of antibiotics known as lincomycin. It inhibits the growth of bacteria by binding to the 50S subunit of the ribosome, which in turn inhibits the bacterial protein synthesis [17]. It confers activity against Gram-positive aerobes and anaerobes and selected Gram-negative anaerobes.

Following clindamycin, ceftazidime was the third most common empirical treatment practised by the two tertiary hospitals with 10.1% of patients receiving the drug. It demonstrates activity against Gram-positive and Gram-negative bacteria [24].

The selection of antibiotics for NF is multi-factorial and includes parameters such as the drug characteristics, effectiveness, safety profile, cost and local multidrug resistance profile, which influences the antibiotic choice. Often the accessibility, cost and effectiveness are taken into consideration by public tertiary hospitals to achieve a maximum benefit with a reasonable expenditure on antibiotics. In this study, a relatively high recovery rate was achieved of 59.4%, emphasising the effectiveness of the empirical antibiotics prescribed. In the cases with a poorer recovery, diabetes was an important comorbidity to be considered because vascular insufficiency and poor tissue penetration in this metabolic disorder negatively impacts the systemic bioavailability of the antibiotic, which in turn can contribute to an increased risk of resistance [25,26].

Given the paucity of studies such as this, the outcome of this study is intended to provide an insight on the current trend of empirical antibiotic prescription, which is intended to improve the management of NF. Reasonable measures were taken to reduce biasness of this study. Randomisation was done to reduce selection bias as people were randomly selected to take part in the investigation. In addition, matching was done to mitigate the confounding bias. One of the limitations was the number of centres involved in this study. Studies in the future should involve more healthcare centres, with consideration of variables such as other comorbidities. Studying the cost involved may also provide a more holistic perspective for clinicians in making prescription decisions in NF, which hopefully will improve the prognosis of the disease.

## 5. Conclusions

Given the changes in epidemiology for NF across the world, clinicians face the challenge of constantly reviewing existing antibiotic prescribing guidelines. The management of NF via antibiotics includes the consideration of multiple factors including the virulence of the pathogens, local multidrug resistance profile, cost and accessibility to antibiotics. In this study, the three main empirical antibiotics that were prescribed weighing these factors were ampicillin + sulbactam, clindamycin and ceftazidime. The patients who received antibiotics had 0.779 times lower chances of having an amputation. The study revealed the impact on the outcome of the disease progression based on the choice of antibiotics and with larger studies in the future, the evidence-based prescription practice for NF can be enhanced to yield the best outcomes for patients.

## Figures and Tables

**Table 1:** Distribution of patients by socio-demographic characteristics.

Variables	Total (*n* = 420)	
	Frequency	Percentage
**Year**		
2014	78	18.6
2015	65	15.5
2016	88	21.0
2017	97	23.1
2018	92	21.9
**Age (Mean ± SD)**	**56.25 ± 12.04**	
<29	3	0.7
30–39	35	8.3
40–49	83	19.8
50–59	133	31.7
60–69	105	25.0
70–79	53	12.6
80–89	6	1.4
90–99	2	0.5
**Gender**		
Male	246	58.9
Female	174	41.4
**Race**		
Malay	277	66
Indian	96	22.9
Chinese	44	10.5
Others	3	0.7

**Table 2:** Association between amputation, the microorganism and the antibiotics used.

Variables	Total (*n* = 420)		
	Frequency	Percentage	*p*-Value
**Types of Microorganisms**			<0.001
*Streptococcus*	79	18.8	
Others	66	15.7	
*Pseudomonas aeruginosa*	61	14.5	
No growth	49	11.7	
*Staphylococcus*	49	11.7	
*Klebsiella pneumoniae*	42	10.0	
*Proteus*	39	9.3	
*Enterococcus*	30	7.1	
Mixed growth	5	1.2	
**Types of Antibiotics**			
Ampicillin + sulbactam	258	61.4	0.23
Others	69	16.4	
Ceftazidime	38	9.0	
Clindamycin	55	13.1	

**Table 3:** Antibiotics and the rate of amputation.

Antibiotics			Amputation		Total
			No	Yes	
Antibiotic	Ampicillin + sulbactam	Count	176	82	258
		%	68.2	31.8	100.0
	Clindamycin	Count	38	17	55
		%	69.1	30.9	100.0
	Ceftazidime	Count	30	11	41
		%	73.2	26.8	100.0
	Ceftriaxone	Count	1	3	4
		%	25.0	75.0	100.0
	Cefuroxime	Count	10	0	10
		%	100.0	0.0	100.0
	Ertapenem	Count	2	1	3
		%	66.7	33.3	100.0
	Trimethoprim/sulfamethoxazole	Count	0	1	1
		%	0.0	100.0	100.0
	Erythromycin	Count	1	0	1
		%	100.0	0.0	100.0
	Penicillin	Count	1	0	1
		%	100.0	0.0	100.0
	Gentamycin	Count	2	0	2
		%	100.0	0.0	100.0
	Cloxacillin	Count	11	0	11
		%	100.0	0.0	100.0
	Metronidazole	Count	0	1	1
		%	0.0	100.0	100.0
	Meropenem	Count	10	7	17
		%	58.8	41.2	100.0
	Oxacillin	Count	2	1	3
		%	66.7	33.3	100.0
	Imipenem	Count	2	0	2
		%	100.0	0.0	100.0
	Piperacillin/tazobactam	Count	7	0	7
		%	100.0	0.0	100.0
	Amoxicillin/clavulanic acid	Count	3	0	3
		%	100.0	0.0	100.0
Total		Count	296	124	420
		%	70.5	29.5	100.0

**Table 4:** Amputation and associated variables.

Variables	Total (*n* = 420)		
	Frequency	Percentage	*p*-Value
**Amputation**			
No	296	70.5	
Yes	124	29.5	
**Age (Mean ± SD)**	**56.25 ± 12.04**		0.73
<29	3	0.7	
30–39	35	8.3	
40–49	83	19.8	
50–59	133	31.7	
60–69	105	25.0	
70–79	53	12.6	
80–89	6	1.4	
90–99	2	0.5	
**Gender**			0.312
Male	246	58.9	
Female	174	41.4	
Race			0.230
Malay	277	66	
Indian	96	22.9	
Chinese	44	10.5	
Others	3	0.7	
**LRINEC Score**	(7.80 ± 2.5)		0.016
**Days of Stay (Mean ± SD)**	(21.7 ± 15.5)		0.012
**Amputation Level**			<0.001
Nil	296	70.5	
BKA	62	14.8	
AKA	31	7.4	
Others (Lower Limb)	29	6.9	
Others (Upper Limb)	2	0.5	

**Table 5:** Distribution of the microorganism and outcomes.

Variable			Amputation		Total
			No	Yes	
**Microorganism**	*Streptococcus*	Count	65	14	79
		%	82.3	17.7	100.0
	*Pseudomonas aeruginosa*	Count	46	15	61
		%	75.4	24.6	100.0
	*Staphylococcus*	Count	46	3	49
		%	93.9	6.1	100.0
	*Klebsiella pneumoniae*	Count	24	18	42
		%	57.1	42.9	100.0
	*Proteus*	Count	21	18	39
		%	53.8	46.2	100.0
	*Enterococcus*	Count	18	12	30
		%	60.0	40.0	100.0
	Others	Count	31	35	66
		%	47.0	53.0	100.0
	No growth	Count	34	15	49
		%	69.4	30.6	100.0
	Mixed growth	Count	4	1	5
		%	80.0	20.0	100.0

**Table 6:** Multiple linear regression analysis for the predictors of the amputation rate.

Variable	Unstandardised Coefficient		Standardised Coefficient	*p*-Value
	B	SE	B	
LRINEC score	−0.934	0.116	1.669	0.009 *
Type of Antibiotic	−0.779	0.115	0.525	0.045 *

Note: Multiple linear regression; (*): significant *p* < 0.05.

## Data Availability

The data used and/or analysed during the current study are available from the corresponding author upon request.
